# Tree Shade Improves Milking Performance, Apparent Digestibility, Antioxidant Capacity, and Immunity of Dairy Cows in Open Sheds

**DOI:** 10.3390/ani15111673

**Published:** 2025-06-05

**Authors:** Jianjie Li, Yinghao Zhou, Man Feng, Lianjie Song, Yuqing Liu, Haitong Yang, Lu Zhang, Ao Zhang, Xinnian Zhao, Xinsheng Sun, Yuhong Gao, Jianjun Guo

**Affiliations:** 1 College of Animal Science and Technology, Hebei Agricultural University, Baoding 071001, China; 18830858605@163.com (J.L.); 15832391785@163.com (Y.L.); 18831227720@163.com (H.Y.); 15512071905@126.com (L.Z.); 13483888591@163.com (A.Z.); zhaoxinnian0704@163.com (X.Z.); 2 Chengde Academy of Agricultural and Forestry Sciences, Chengde 067000, China; mymyinghao@163.com (Y.Z.); fengman59@163.com (M.F.); songlianjie1993@163.com (L.S.); 3 College of Information Science and Technology, Hebei Agricultural University, Baoding 071001, China; 2002444908@sohu.com

**Keywords:** dairy cow, shade, lactation performance, antioxidant capacity, immunity

## Abstract

Dairy cows are suffering from heat stress (HS) worldwide, and this has become a continual challenge in dairy production systems. The objective of this study was to investigate the effects of the shade of poplar trees on alleviating HS via measuring milk production, nutrient digestibility, immunity, and antioxidant capability in the serum of dairy cows in open cowsheds. A total of 540 lactating Holstein cows were assigned to 2 groups (3 cowsheds per group, 90 cows per cowshed), including the treatment group with poplar trees on the west side of sheds and the control group without trees. This study was carried out in the early-hot season, hot season, and late-hot season. The results showed the following: (1) During the hot season, shaded cows exhibited lower respiration rate (*p* < 0.05) and higher dry matter intake (*p* < 0.05) and daily milk yield (*p* < 0.05), compared with the control. Also, apparent digestibility of crude protein, acid detergent fiber, and neutral detergent fiber demonstrated an increase (*p* < 0.05) in shaded cows during the hot season. (2) Total antioxidant capacity and superoxide dismutase activity in the serum of shaded cows increased (*p* < 0.05) during the hot season and late-hot season, compared with the control. (3) Serum interleukin-4, immunoglobulin G, and immunoglobulin M concentrations of shaded cows were greater (*p* < 0.05) than the control during the hot season. Additionally, serum concentrations of HSP60, HSP70, and HSP90 in shaded cows showed a decrease (*p* < 0.05) during the hot season. In conclusion, shade from poplar trees can mitigate the adverse impact of high-temperature environments on cows in open cowsheds.

## Simple Summary

In recent years, open cowsheds have been widely used for dairy cows, and the western exposure to the afternoon sun has become a serious problem in the high-temperature season. Mitigating heat stress in dairy cows has become a continual challenge in dairy production systems. The objective of this study was to investigate the effects of the shade of poplar trees planted along the west side of open sheds on production performance in cows. The results showed that shading reduced respiration rate, improved lactation performance, and elevated antioxidant activity of cows during the hot season. Immune functions involving interleukins, immunoglobulins, and heat stress proteins were also enhanced by shade provision. Thus, the shade of poplar trees can effectively prevent the HS-related impact of afternoon sun during the hot season.

## 1. Introduction

Global climate change in recent years has elevated the heat-duration exposure of Holstein dairy cows in China, and the increasing risk from heat stress (HS) has caused dairy producers to be concerned. Dairy cows, as cold-resistant and heat-labile animals, have generally optimal milking performance in a temperature range from 5 °C to 18 °C [1,2]. When the ambient temperature surpasses the upper critical threshold for dairy cows, a disruption occurs in the balance between body heat production and dissipation [3]. This thermal imbalance triggers a series of adaptive physiological responses as the cows attempt to maintain homeothermy [4]. Numerous studies have corroborated the specific physiological manifestations of HS in dairy cows, e.g., the increases in rectal temperature, respiratory rate, and saliva production [5]. Previous studies have demonstrated that feed intake of dairy cows and lactation performance, including milk yield and milk quality, are negatively affected under the conditions of HS exposure [6,7,8]. Particularly for high-yielding dairy cows, the cumulative exposure to HS significantly impacts the milking performance [9]. Gunn et al. [10] predicted that milk loss due to HS will increase at a rate of 174 kg per cow per decade in the 21st century. Thus, it is necessary to minimize the adverse responses to HS of dairy cows, which has become a continual challenge in many countries worldwide.

Although some indoor cooling facilities (fan, sprinkler, wet curtain cooling system, etc.) have been adopted to mitigate the incidence of HS in dairy cows in summer, a substantial amount of consumed water causes a high-humidity environment in cowsheds and leads to more difficulty in wastewater treatment [11,12]. A published review indicated that shade provision contributes to reducing heat load for cattle, e.g., the mitigation of physiological responses involving respiration rate, body temperature, and panting score, the increase in growth performance (weight gain and feed efficiency), and improvement in behavioral patterns [13]. Additionally, the impacts of shade on cows exposed to HS depend on shade structures and materials. Shade design and its implementation would have some potential in the development of livestock husbandry because they do not rely on extra resource inputs involving electricity and water consumption. Recent studies have reported that many resources for shade structures such as trees could be available, and their effectiveness and economic benefits should be explored [14]. Published studies showed that high trees (e.g., paulownia and poplar) have shading effects similar to sunshades, while well-designed sunshades will reduce heat load in dairy cows or cattle by 30% to 50%, as reflected by a normal rectal temperature and respiratory rate [15]. However, it has also been seen that cows prefer natural shade provided by trees to protect themselves against solar radiation rather than other structures of shades [16]. A report on shaded beef cattle demonstrated that the tree shades reduced the concentration of cortisol in the hair of cattle during summer. This alleviates the negative impact of HS on average daily gain (ADG) and, thus, improves production performance [17]. In recent years, open and semi-open cowsheds have been widely used for dairy cows in China, and western exposure to the afternoon sun, which easily causes indoor overheating, has become a serious problem in the high-temperature season [18]. Therefore, the objectives of the present study were to investigate the influences of shade provided by poplar trees planted along the west side of cowsheds on milking performance, physiological properties, immunity, and antioxidant capability under heat conditions, which would provide insights into preventing HS in dairy cows.

## 2. Materials and Methods

### 2.1. Preparation of Experimental Cowsheds

The protocols (2023048) were approved by the Animal Care Committee in Hebei Agricultural University in accordance with the University’s guidelines for animal research.

**Figure 1:**
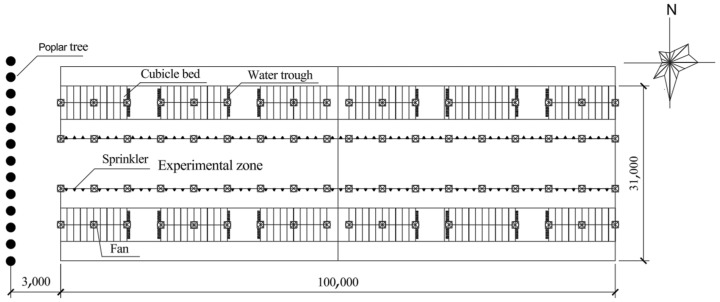
Plan layout of the cowshed. In the shaded group, a row of 15 m high poplar trees were planted along the west side of the cowshed, and the spacing between adjacent trees was 3 m.

The present study was carried out on a commercial dairy farm in Hebei province in China, which is located at 114.98° E and 38.74° N. In this location, the solar elevation angle gradually increases from May to June, reaching the annual maximum of approximately 75° at 1200 to 1300 h on the summer solstice day. After the summer solstice, the solar elevation angle gradually decreases to approximately 36° at 1200 to 1300 h on 30 October. The open cowsheds (100 m length, 31 m span, 5.5 m high eave, 31 m high roof ridge) with south–north orientation were used, and the architectural structures of the roof were made of steel sandwich panels with polystyrene foam (10 cm thickness). The west half of the cowsheds was used for the experimental zone. The plan arrangement of the cowshed is shown in Figure 1.

### 2.2. Experimental Design

Two experimental groups (the control and shaded group) were contained in this study, with three cowsheds per group. A row of 15 m high and 5-year-old poplar trees (6 m crown height) were planted nearly 3 m away from the west side of each cowshed in the shaded group to reduce exposure to the afternoon sun. The row with 13 trees for each cowshed was 36 m long, and the spacing between adjacent trees was approximately 3 m. According to the method reported by Wang Hui et al. [19], during the noon period of 1200 to 1300, the average shaded area of each row of trees is 440 m^2^ in the early-hot season (May), 410 m^2^ in the hot season (June to August), and 1010 m^2^ in the late-hot season (September to October). After the summer solstice day, as time goes by, the shade gradually lengthens and the shaded area becomes larger. At 1600, the shaded areas for three stages mentioned above are 1529 m^2^, 1394 m^2^, and 2915 m^2^, respectively. The cowsheds in the control group were of the same size as the shaded group but without trees. The total of 540 lactating Holstein cows (560 ± 12 kg BW, 185 ± 25 DIM, 31.0 ± 1.25 kg milk yield per day, 2.5 ± 0.5 parities) were weighed using a weighing scale at the start of experiment and randomly assigned into two groups. The entire experiment lasted for 5 months, and was divided into three stages, including the early-hot season (average 27.2 °C, humidity 75%, wind speed 2.8 m/s, solar radiation 865 W/m^2^·d), hot season (average 29.5 °C, humidity 80%, wind speed 2.1 m/s, solar radiation 873 W/m^2^·d), and late-hot season (average 15.2 °C, humidity 65%, wind speed 1.9 m/s, solar radiation 671 W/m^2^·d)).

All experimental cowsheds implemented a cooling procedure via the combination of sprayers and fans. When the ambient temperature in the control sheds exceeded 25 °C, the fans in all experimental sheds were turned on, and when the temperature exceeded 27 °C, the spraying system came into operation in an intermittent mode (spraying for 1 min and pausing for 9 min). All cows were offered similar total mixed ration in a ratio of concentrate and roughage of 45:55 (DM basis) three times daily at approximately 0600, 1200, and 1800 h. The ration was formulated to meet the nutritional requirements for dairy cows (NRC, 2001) [20]. The ingredients and nutrient composition of the ration are listed in Table 1. The drinking water and feed were provided at libitum throughout the experiment. All cows were milked three times daily at approximately 0530, 1130, and 1730 h in a milking parlor using an automatic milking system (9JFTA-64B, Zibo, Shandong, China) for automatic recording of individual cow milk yield of each milking. During the entire experimental period, ambient temperature and relative humidity in the sheds were monitored at intervals of 1 h using temperature and humidity recorders (Model KTH-350-I, Montreuil, France) to obtain the curves of temperature and humidity changes.

### 2.3. Physiological Parameters Measurement

The physiological parameters included respiratory rate, rectal temperature, and skin temperature. Fifteen cows in each shed were used to examine these parameters during the period from 1200 to 1300 h for the last three days in each month from May to October, and the above three parameters for each experimental stage were analyzed. The respiratory rate was measured by a traditional method via recording the number of breaths per minute using a counter when the cows were in a resting state [21]. The rectal temperature was measured using a veterinary thermometer, which was inserted into the anus to 4 to 5 cm depth and kept there for 3 to 5 min. The skin temperatures of torso, four limbs, neck, and ears were measured using an infrared thermal imager (Testo-890, Lenzkirch, Germany) and calculated as described by Yan et al. [22].

### 2.4. Milk Sampling and Analysis

Milk samples (40 mL) from each cow were collected at each milking and mixed samples from the same day (morning, noon, and night, 4:3:3 by volume) for the last three days in each month from May to October. The samples were kept at 4 °C with an added preservative of 2-bromo-2-nitro-1,3-propanediol until analysis for milk quality [23]. The parameters of milk quality involving milk fat, lactose, protein, urea nitrogen, and somatic cell count were measured using a milk analyzer (Milko-Scan^TM^ Mar, Hillerød, Denmark) in DHI center of Breeding Livestock and Poultry Quality Monitoring Station, Hebei province, China. The 4% fat-corrected milk (4% FCM) was calculated using the following equation reported by Tyrrell and Reid (1965) [24].

4% FCM (kg/d) = [0.4 × milk yield (kg/d)] + [15 × milk fat yield (kg/d)]

### 2.5. Feed and Fecal Sampling and Analysis

The feed provided to cows and the refusals were weighed daily throughout the experimental period, and the dry matter intake (DMI) was analyzed for each experimental stage. The fresh feces from 15 cows in each shed were collected by hand via rectal palpation for the last three days in each natural month from May to October [25]; meanwhile, the feed and refusal samples were also collected. One aliquot of collected fecal samples for crude protein (CP) measurement was added with hydrochloric acid (10%, vol/vol) to prevent fecal nitrogen from volatilizing, with the addition of 10 mL acid per 100 g feces; the other equal aliquot was not supplemented with hydrochloric acid for measurement of the other nutrients (DM, ether extract (EE), acid detergent fiber (ADF), neutral detergent fiber (NDF), calcium (Ca), and phosphorus (P)) [26]. All fecal samples were kept at −20 °C until analysis.

The feed and fecal samples were dried at 55 °C for 48 h and then ground using a grinder (Model SL-250, Yongkang, Zhejiang, China) to pass through a 1 mm screen for further measurements of DM, CP, EE, Ca, and P contents, according to the methods reported by Association of Official Analytical Chemists (AOAC International, 2005) [27]. The NDF and ADF contents were measured using the method from Van Soest et al. [28]. The apparent digestibility of nutrients mentioned above was analyzed using the approach of acid-insoluble ash as an internal marker [29].

### 2.6. Blood Sampling and Measurement

#### 2.6.1. Blood Sampling

For blood samplings (15 cows/shed), the cows were fasted overnight each month on the last night from May to October, and the 10 mL blood from each cow was collected into evacuated tubes without anticoagulant from the tail vein [30,31]. The blood samples were kept at 4 °C for 30 min and then centrifuged at 3000× *g* for 15 min at 4 °C to collect serum. One aliquot of serum (2 mL) was preserved at −20 °C until analysis for the antioxidant enzymes activity and immunity. The rest of the aliquot (1 mL) was immediately added with RNAstore (Thermo Fisher, Waltham, MA, USA) to prevent RNA from degradation and then stored at −80 °C until further analysis of mRNA expression of the genes sensitive to heat load.

#### 2.6.2. Serum Biochemistry

The activities of total antioxidant capacity (T-AOC), superoxide dismutase (SOD), glutathione-S-transferase (GSH-ST), and malondialdehyde (MDA) were detected by spectrophotometry using an ELISA kit (Nanjing Jiancheng Bioengineering Institute, Nanjing, China) as described in the manufacturer’s protocol. The contents of T-AOC, SOD, and GSH-ST were determined by the colorimetry method, and MDA content was determined by the thiobarbituric acid method [32]. The immune responses were evaluated by a panel of parameters involving interleukins (IL-4 and IL-6), immunoglobulins (IgA, IgG, and IgM), and heat shock proteins (HSP60, HSP70, and HSP90). The measurements of immune parameters mentioned above were implemented by the quantitative sandwich ELISA technique using the DR-200BS enzyme-labeled instrument (Wuxi Huawei Delang Instrument Co., Ltd., Wuxi, China) [33] and ELISA kit (Nanjing Jiancheng Bioengineering Research Institute, Nanjing, China; intra-assay variation, all <10%).

#### 2.6.3. Gene Expression

Among the serum biochemical parameters mentioned above, those parameters that showed sensitivity to the shade treatment were chosen for a more in-depth examination of gene expression. The primer sequences of selected genes, shown in Table 2, were designed using Premier 8.0 software (Premier, Markham, ON, Canada).

The total RNA (tRNA) from the serum samples was extracted using an RNA Blood Kit (LS1040, Promega, Shanghai, China). The quality of the separated tRNA was examined by the gel electrophoresis test and the ratio of OD_260nm_ to OD_280nm_. Only when the electrophoretic band is very integral without any sign of degradation and the value of OD_260nm_/OD_280nm_ ranges from 1.8 to 2.0 is the tRNA available for further reverse transcription. According to the instructions of the iScript Reverse Transcription kit (Bio-Rad, Hercules, CA, USA), the first-strand cDNA was produced by the procedure at 37 °C for 15 min and then 85 °C for 5 s. A SybrGreen-based quantitative PCR (qPCR) reaction was proceeded to determine the relative quantification of mRNA expression for the genes mentioned above by 7900 HT Fast Real-time PCR System (Applied Biosystems, Foster, CA, USA), according to the protocol of GoTaq qPCR Master Mix A 6001 kit (Promega, Madison, WI, USA). Briefly, a 20 µL of PCR reaction system, including 2 µL of cDNA, 1.2 µL of 10 µM primers, 10 µL of 2×SuperReal PreMix Plus, 0.4 µL of 50×ROX Reference Dye, and 6.4 µL of RNase-free ddH_2_O, was performed for each gene. The qPCR procession began with an initial denaturation reaction (95 °C, 10 min), followed by 40 cycles of amplification: 95 °C for 10 s for denaturation, 60 °C for 1 min for annealing, and 95 °C for 15 s for extension. Finally, a melting curve was generated from 60 °C to 99 °C. The relative quantification of target genes was analyzed by the 2^−ΔΔCt^ method from Kumar et al., where the amplification data were normalized to GAPDH [34], because the expression level of GAPDH remains relatively stable in most experimental conditions [35,36,37,38].

### 2.7. Statistical Analysis

The data were analyzed by one-way analysis of variance (ANOVA) using SAS 9.2 software (SAS Institute Inc., Cary, NC, USA). A statistical method of t-test was used to compare the means of two groups (the control and shaded group). The data were checked for normality and homogeneity of variance prior to analysis by t-test. The differences were considered significant at *p* ≤ 0.05, and tendencies were declared at 0.05 < *p* ≤ 0.10. The data are expressed as means with standard error of the mean (SEM).

## 3. Results

### 3.1. Changes of Ambient Temperature and Relative Humidity in Cowsheds

**Figure 2:**
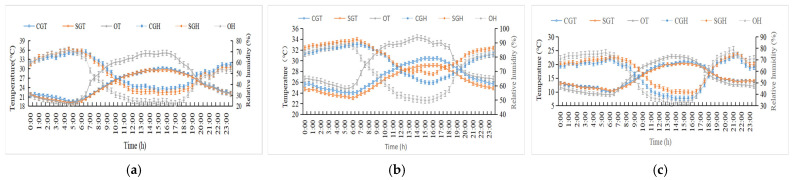
Changes in the temperature and relative humidity in cowsheds. (**a**) Early-hot season. (**b**) Hot season. (**c**) Late-hot season. CGT: control group temperature; SGT: shaded group temperature; OT: outdoor temperature; CGH; control group humidity; SGH: shaded group humidity; OT: outdoor humidity.

The changes in ambient temperature and relative humidity for the three stages are shown in Figure 2. During the hot season, the temperature ranged from 19.5 °C to 30.1 °C (average 24.9 °C) in the control sheds and 19.3 °C to 29.6 °C (average 24.6 °C) in the shaded sheds. Particularly, during the period of 0900 to 2000, the temperature in the shaded sheds was lower (*p* < 0.05) by 1.13 °C compared to the control sheds. However, no difference (*p* > 0.05) in temperature was observed between the two groups during either the early- or late-hot seasons. Additionally, the indoor relative humidity for each stage had no difference (*p* > 0.05) between the two groups.

### 3.2. Effects of Shade on Physiological Parameters and Milking Performance in Dairy Cows

**Figure 3:**
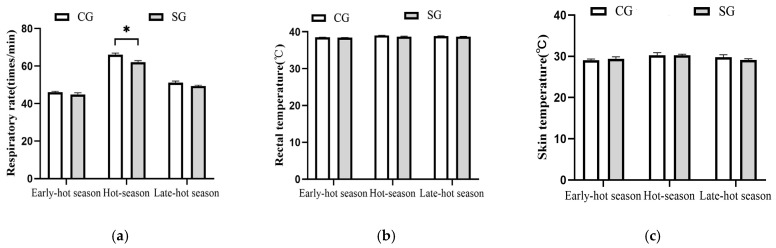
Effects of tree shades on physiological parameters in dairy cows. (**a**) Respiratory rate. (**b**) Rectal temperature. (**c**) Skin temperature. CG: control group; SG: shaded group; * *p* < 0.05.

The effects of shade on the physiological parameters in dairy cows are shown in Figure 3. During the hot season, the respiratory rates of dairy cows in the shaded group were reduced (*p* = 0.03) by 6.14% compared with the control group (62.07 vs. 66.13, Figure 3a), while the other two parameters (rectal temperature and skin temperature) demonstrated no difference (*p* > 0.05) between the two groups (Figure 3b,c). For early- and late-hot seasons, the shade provision resulted in no difference (*p* > 0.05) for the three parameters mentioned above compared to the control.

The effects of shade on the milk yield and quality are given in Table 3. During the hot season, the DMI, milk yield, and 4% FCM in the shaded group increased by 8.85% (*p* = 0.02), 5.12% (*p* = 0.04), and 5.18% (*p* = 0.03), respectively, compared with the control. Additionally, when the cows were provided with the shade from trees, the urea nitrogen content demonstrated a decrease of 12.45% (*p* = 0.03) compared with the control. No difference (*p* > 0.05) was observed between the two groups in milk yield or milk composition during the early- and late-hot seasons.

### 3.3. Effects of Shade on Apparent Nutrient Digestibility in Dairy Cows

The effects of shade on the apparent digestibility of nutrients were analyzed, and the results are shown in Table 4. During the early- and late-hot seasons, there was no difference (*p* > 0.05) in the apparent digestibility of all nutrients between the two groups. However, during the hot season, the shade-provided cows demonstrated an increase in the CP (*p* = 0.03), NDF (*p* = 0.02), or ADF (*p* = 0.02) digestibility compared with the control cows, while the digestibility of other nutrients showed no difference (*p* > 0.05) between the two groups.

### 3.4. Effects of Shade on Antioxidant Enzyme Activities in Serum of Dairy Cows

The activities of a panel of antioxidant enzymes (T-AOC, SOD, GSH-ST, and MDA) in serum are listed in Table 5. The activities of enzymes mentioned above demonstrated no difference (*p* > 0.05) between the two groups in the early-hot season. However, during the hot season, both SOD and T-AOC activities in the shaded group demonstrated differences (*p* < 0.05) between the two groups, exhibiting an increase of 17.80% (*p* = 0.02) in SOD and 16.15% (*p* = 0.02) in T-AOC, compared with the control. Particularly, the SOD activity was also influenced (*p* = 0.02) by the shade during the late-hot season, increased by 12.63% in the late-hot season compared with the control.

### 3.5. Effects of Shade on Immune Property in Serum of Dairy Cows

The effects of shade on the immune parameters involving immunoglobulins, interleukins, and HSPs are shown in Table 6 and Figure 3. During the early-hot season, all immune parameters in the serum were not affected (*p* > 0.05) by the shade. However, when the cows were raised through the hot season, the serum concentrations of IgG, IgM, and IL-4 in the shaded group increased by 18.06% (*p* = 0.04), 19.26% (*p* = 0.04), and 9.17% (*p* = 0.03), respectively, compared with the control. During the late-hot season, the serum IL-4 concentration for the shade-provided cows also increased by 20.83% (*p* = 0.03) compared with the control, while the other immune parameters involving IgA, IgG, IgM, and IL-6 demonstrated no difference (*p* > 0.05) between the two groups.

**Figure 4:**
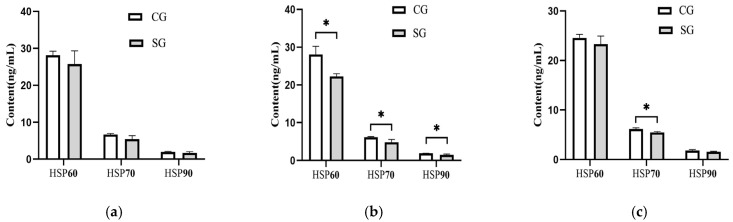
Effects of shade on heat stress protein concentration in serum of dairy cows. (**a**) Early-hot season. (**b**) Hot season. (**c**) Late-hot season. HSP60; Heat shock protein-60; HSP70; Heat shock protein-70; HSP90; Heat shock protein-90; CG: control group; SG: shaded group; * *p* < 0.05.

The serum concentrations of three HSPs in this study were also influenced (*p* < 0.05) by the shade provision during the hot season (Figure 4). The shade-provided cows demonstrated a lower level of 22.10% in HSP60, 20.32% in HSP70, and 20.21% in HSP90 during the hot season than those in the control cows. Particularly, the HSP70 concentration in the shaded group also demonstrated a decrease of 11.67% (*p* = 0.02) during the late-hot season, while no difference (*p* > 0.05) was observed between the two groups for the HSP60 or HSP90 concentration.

### 3.6. Effects of Shade on Gene Expression in Serum of Dairy Cows

**Figure 5:**
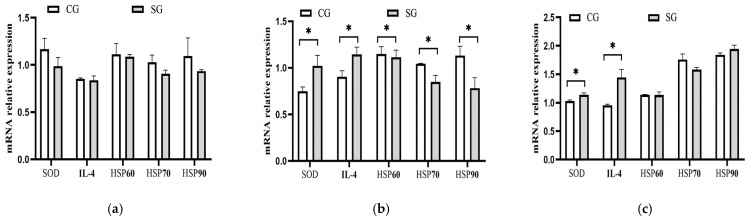
Effects of shade on mRNA expression of related genes in serum of dairy cows. (**a**) Early-hot season. (**b**) Hot season. (**c**) Late-hot season. SOD; Superoxide dismutase; IL-4; Interleukin-4; HSP60; Heat shock protein-60; HSP70; Heat shock protein-70; HSP90; Heat shock protein-90. CG: control group; SG: shaded group; * *p* < 0.05.

According to the serum levels of the antioxidant enzymes and immune parameters measured in this study, five sensitive parameters to heat load, including SOD, HSP60, HSP70, Hsp90, and IL-4, were selected, and their mRNA expression levels were determined. These results are listed in Figure 5. No difference (*p* > 0.05) was observed for each gene between the two groups in the early-hot season. During the hot season, the serum mRNA expressions of HSP70 (*p* = 0.04) and HSP90 (*p* = 0.02) for the shade-provided cows were downregulated by 19.05% and 28.95%, respectively, compared with the control. Meanwhile, the mRNA expressions of SOD and IL-4 in the shaded group were upregulated by 39.19% (*p* = 0.02) and 26.67% (*p* = 0.02) during the hot season, respectively. During the late-hot season, the expression levels of SOD and IL-4 were also upregulated by 10.68% (*p* = 0.02) and 51.58% (*p* = 0.03), respectively.

## 4. Discussion

### 4.1. Effects of Tree Shades on Physiology Properties in Dairy Cows

The aim of this study was to evaluate the effects of shade from high trees on the physiological properties, milking performance, physiological properties, antioxidant capability, and immunity so as to protect dairy cows against the afternoon sun exposure in the hot season. When dairy cows are exposed to HS conditions, the physiological responses (e.g., respiratory rate and rectal temperature) usually occur to defend against HS before production performance is influenced, which means that the physiological parameters are very sensitive to heat exposure. In this study, the respiratory rates of the unshaded cows, as an important physiological indicator, were greater by 6.14% than the shaded cows. The majority of published work has also demonstrated that dairy cows exposed to high-temperature environments firstly enforce heat dissipation to keep internal heat balance by increasing respiratory frequency [39,40,41]. The decrease in respiratory rates in the shaded groups can be attributed to the high trees planted along the west side of cowsheds because they can block out direct and indirect solar radiation from the afternoon sun in the hot season. The indoor temperature in the shaded group decreased by 1.13 °C during the period of 0900 to 2000 in the hot season, indirectly reflecting the positive effect of the shade on reducing thermal radiation. It was proved by a previous study from Hong et al. [42] that the indoor temperature is closely related to the thermal radiation of the roof. It is worth noting that the species of the planted tree in this study was a 15 m high poplar tree. Its trunk was approximately 9 m high, which was higher than the 5.5 m high eave of the cowshed. Therefore, the poplar trees cannot hinder outdoor wind into the cowsheds, and do not compromise the cooling effects of tree shades.

### 4.2. Effects of Tree Shades on Milking Performance and Apparent Digestibility in Dairy Cows

When dairy cows experience a long-lasting HS, whether mild or serious, production performance (feed intake, milk yield, and milk quality) will be negatively affected, particularly DMI [43,44]. Generally, in high-temperature environments, decreasing DMI is a protective mechanism to maintain the heat balance by regulating metabolic rate and heat production. The lower DMI contributes to reducing the metabolic rate to produce less heat to prevent heat load; however, the energy used for production will also be reduced, resulting in a decrease in lactation performance. Early studies indicated that grazing cows exposed to the direct solar radiation decreased the DMI, and the milk yield loss could averagely reach 15%, and up to 40%, under an extreme HS condition [45]. In recent studies, whether using natural or artificial shade, the shade provision may avoid direct solar radiation and then improve the comfort of dairy cows in the hot summer, which is associated with the improvement in production performance (e.g., DMI, milk yield, and milk composition) [46]. Our present results also demonstrate that the shade from the high trees could effectively relieve the heat effects of the afternoon sun during the hot summer, exhibiting that the DMI and milk yield of the shade-provided cows increased by 8.85% and 5.12%, respectively, compared with the unshaded cows. This agrees with Bucklin et al., who reported that lactating cows provided with a sunshade in the hot summer increased DMI by up to 20% and milk production by 12% [47]. In addition, the high-temperature conditions greatly impacted the milk quality of dairy cows, which was proved by Abreu et al. [48]. In our experiment, dairy cows in the shaded group demonstrated an increased tendency in milk protein and a significant decrease in milk urea nitrogen compared with the control group. It might be explained that the shading can mitigate the adverse impacts of HS on cows. Previous reports indicated that the heat environment influenced the microbiota structure in the rumen or gut, and affected the digestion and fermentation of feed and the synthesis of certain nutrients [49]. Thus, it is speculated that the shades in our study may mitigate the negative effect of a high-temperature environment on the protein use efficiency during the hot season and reduce the nitrogen waste. Our present data show that the apparent digestibility of CP for the shade-provided cows increased by 8.18% compared to the unshaded cows. Moreover, the digestibility of NDF and ADF in the shaded group was also found to be greater than those in the unshaded group. The disparity can be attributed to the physiological responses of the organisms under a heat environment. A large amount of blood is distributed to the body’s surface to help dissipate heat, resulting in a decrease in blood flow to the digestive tract, which leads to a reduction in the apparent digestibility of nutrients. These findings in our study highlight the positive impact of the shading on the digestive efficiency of cows, providing valuable insights into the management of dairy cows in hot environments.

### 4.3. Effects of Tree Shades on HSPs and Antioxidant Capacity in Dairy Cows

It is well documented that when animals are exposed to high-temperature stress, an increase in the concentrations of serum HSPs (HSP60, HSP70, HSP90, etc.) occurs, accompanied by upregulation of their gene expression [50]. This biological response is a crucial self-protective mechanism. High-temperature environments can cause proteins within cells to denature, losing their normal three-dimensional structures and functions. The elevated levels of HSPs serve to recognize, bind to, and refold these denatured or abnormal proteins, preventing their aggregation and restoring their native conformations. Our present findings on the three HSPs mentioned above agree with the previous results from Bagath et al. [51]. When cows were raised in the shaded sheds, the three HSPs concentrations mentioned above significantly decreased compared with the unshaded sheds in this case. In particular, for HSP70, both the protein content and its mRNA level for the shade-provided cows were lower than those for the unshaded cows in the hot season, and these lower levels were also found in the late-hot season. Similar studies on HSP70 induced by HS in animals have recently been published [52,53].

In addition, a recent study from Arnal et al. indicated that HSP70 can be closely related to the antioxidant enzyme system [54]. It was suggested that HSP70 can reduce the generation of reactive oxygen species by activating the antioxidant enzymes system to defend against oxidative damage. In the present study, we measured a panel of antioxidant enzymes to assess the physiological status of cows. The results revealed that, during the hot season or late-hot season, the shade-provided cows exhibited higher activities of SOD and T-AOC compared to the unshaded cows. The elevated activities of SOD and T-AOC in the shaded cows reflected an increase in oxidative stress. It is likely that the shade created a relatively cooling microenvironment, enabling the cows’ antioxidant defense systems to respond more actively to HS [55,56]. As such, the shade can serve as a crucial factor in helping cows resist oxidative stress, such as the accumulation of free radicals, which is induced by the harsh thermal environment.

### 4.4. Effects of Tree Shades on Immunity in Dairy Cows

The results from an early study suggested that HSPs may have some association with the generation of interleukins, e.g., HSP70 can promote the induction of some proinflammatory interleukins through the MyD88/Irak/NF-kappaB signal transduction pathway [57]. However, our present data show that the anti-inflammatory factor IL-4 in the shaded group was significantly affected, compared with the control group, exhibiting an increase by up to 28.83% in the serum concentration and by up to 51.6% in the gene expression in the hot and late-hot season. It was reported that IL-4 can enhance the phagocytic activity of macrophages and induce the proliferation and differentiation of B lymphocytes, which contributes to the generation of immunoglobulin [58]. The immunoglobulins, mainly including IgA, IgG, and IgM, are known to possess antibody-like activity, and play crucial roles in immune regulation [59]. In this study, the serum IgG and IgM concentrations increased when cows were provided the tree shade, particularly the IgG concentration, with an increase of 43.15%. These results suggest that the immune function was enhanced by tree shades under the HS conditions. This finding agrees with Xu et al. [60], who reported that the increase in the IgG concentration enhanced animals’ immune function, and it is also deemed that the IgG could be transferred from blood to the mammary gland, which is related to milk synthesis. Our present results involving HSPs, antioxidant activities, and immunity are consistent with the results of lactation performance in this experiment. Based on these findings, the provision of shade to dairy cows would, indeed, have a more beneficial and economical consequence in alleviating HS.

In practical implications, the trees planted within the cow farms serve a dual purpose of aesthetic enhancement and heat load mitigation. Nevertheless, when selecting tree species, their seasonal characteristics must be taken into account. It is essential to choose suitable tree species (poplar tree, pagoda tree, paulownia, etc.) whose leaves fully fall during the cold season, ensuring that the maximum amount of sunlight can penetrate into the indoor areas during winter.

## 5. Conclusions

The shade from poplar trees improved the production performance of dairy cows, demonstrating an increase by 8.79% in DMI and 5.12% in milk yield during the hot season compared to the unshaded cows. Physiologically, the shaded cows’ respiration rates decreased by 8.35%, suggesting that the shading mitigates HS. In terms of immunity and antioxidant capacity, the serum IL-4, IgG, and IgM levels and serum SOD and T-AOC activities increased significantly by the tree shades, while the serum concentrations of HSP60, HSP70, and HSP90 decreased. Our results indicate that the shade of poplar trees along the west side of cowsheds can effectively prevent the HS-related impact of the afternoon sun during the hot season.

## Figures and Tables

**Table 1:** Ingredient and chemical composition of basal diets (DM basis).

Ingredient	% of DM	Chemical Composition	% of DM
Whole corn silage	40.62	NE_I_, MJ/kg	6.75
Oaten hay	15.39	CP	14.08
Steam flaked Corn	14.41	NDF	40.43
Soybean meal	12.85	ADF	25.40
Soybean hull	11.55	Ca	0.64
Cotton meal	3.90	P	0.30
Limestone	0.10		
CaHPO_4_	0.15		
NaCl	0.50		
NaHCO_3_	0.15		
Premix ^(1)^	0.45		
Total	100.00		

^(1)^ Premix was provided by the following (per kg of feed): VA: 400,000 IU; VD3: 150,000 IU; VE: 7000 IU; Cu: 1000 mg; Fe: 5000 mg; Zn: 4500 mg; Mn: 5000 mg; Co: 40 mg; I: 100 mg; Se: 20 mg.

**Table 2:** Primer sequences for PCR amplification of the target genes.

Genes	Primer	Sequences(3′→5′)
SOD	Forward	TGCAGGTCCTCACTTTAATCC
	Reverse	CAGCGTTGCCAGTCTTTGT
HSP60	Forward	GTAGCCGTTACTATGGGG
	Reverse	TCCTTGGCAATAGAGCGT
HSP70	Forward	GACAAGTGCCAGGAGGTGATT
	Reverse	AGTCTGCTGATGATGGGGTTA
HSP90	Forward	CCAGTACATGGAGGGCTTCA
	Reverse	TCCTCTTCCTCGTATTCCTTCA
IL-4	Forward	AGTGCTGGTCTGCTTACTGG
	Reverse	CTTTCTCGTTGTGAGGATGT
GAPDH	Forward	AGGGCTGCTTTTAATTCTGGC
	Reverse	TGACTGTGCCGTTGAACTTGC

SOD: Superoxide Dismutase; HSP60: Heat Shock Proteins 60; HSP70: Heat Shock Proteins 70; HSP90:Heat Shock Proteins 90; IL-4: Interleukin-4; GAPDH: Glyceraldehyde-3-Phosphate Dehydrogenase.

**Table 3:** Effects of shade on the milking performance in dairy cows.

Items	Early-Hot Season				Hot Season				Late-Hot Season			
	CG	SG	SEM	*p*-Value	CG	SG	SEM	*p*-Value	CG	SG	SEM	*p*-Value
Dry matter intake, kg	20.30	20.49	0.16	0.32	18.42	20.05	0.24	0.02	22.28	22.79	0.32	0.18
Milk yield, kg	31.53	31.61	0.43	0.87	28.71	30.18	0.04	0.04	31.48	31.77	0.63	0.67
4% fat-corrected milk, kg	27.71	27.93	0.40	0.59	28.19	29.65	0.08	0.03	28.54	28.50	0.50	0.95
Milk composition												
Fat, %	3.19	3.23	0.02	0.08	3.87	3.89	0.18	0.92	3.38	3.31	0.03	0.06
Protein, %	3.23	3.27	0.07	0.66	3.25	3.37	0.09	0.24	3.33	3.29	0.03	0.30
Lactose, %	5.35	5.35	0.03	1.00	5.25	5.23	0.02	0.45	5.14	5.10	0.02	0.20
Total solid, %	13.74	13.49	0.39	0.57	17.42	17.29	0.08	0.19	13.49	13.25	0.11	0.10
Somatic cell count, ×10^4^ ml	7.11	6.52	0.89	0.54	20.70	20.26	0.84	0.63	9.11	8.07	1.05	0.38
Urea nitrogen, mg/dl	10.73	9.73	0.18	0.07	11.08	9.70	0.42	0.03	7.94	7.76	0.17	0.35
Final days in milk	217	219	4.32	0.67	274	268	3.64	0.23	240	247	3.59	0.14

CG: control group; SG: shaded group; SEM: standard error; *p*: probabilities.

**Table 4:** Effects of shade on the apparent digestibility of nutrients in dairy cows.

Items	Early-Hot Season				Hot Season				Late-Hot Season			
	CG	SG	SEM	*p*-Value	CG	SG	SEM	*p*-Value	CG	SG	SEM	*p*-Value
Dry matter, %	76.0	76.4	0.50	0.47	74.5	75.4	0.42	0.24	75.6	77.0	0.94	0.23
Crude protein, %	75.6	75.6	0.84	0.91	65.7	68.0	0.72	0.03	75.2	76.0	0.50	0.25
Ether extract, %	77.7	77.1	0.49	0.29	73.8	74.4	0.42	0.26	77.8	77.6	0.91	0.86
Neutral detergent fiber, %	65.7	66.4	0.56	0.33	60.0	64.5	1.11	0.02	63.1	66.5	1.22	0.06
Acid detergent fiber, %	53.3	52.1	0.56	0.11	44.1	49.0	1.22	0.02	51.6	54.3	1.70	0.18
Calcium, %	32.7	32.7	0.50	0.94	28.1	29.2	0.46	0.08	31.8	32.3	0.99	0.69
Phosphorus, %	56.3	55.6	0.44	0.18	51.7	52.2	0.31	0.21	53.9	54.3	0.66	0.58
Ash, %	44.0	45.7	0.68	0.06	39.8	41.7	0.92	0.11	41.5	43.0	0.93	0.17

CG: control group; SG: shaded group; SEM: standard error; *p*: probabilities.

**Table 5:** Effects of shade on the antioxidant enzymes activity in serum of dairy cows.

Antioxidant Parameters	Early-Hot Season				Hot-Season				Late-Hot Season			
	CG	SG	SEM	*p*-Value	CG	SG	SEM	*p*-Value	CG	SG	SEM	*p*-Value
T-AOC, U/ml	11.84	12.16	0.67	0.65	7.81	9.20	0.36	0.02	6.60	7.42	0.44	0.14
SOD, U/ml	71.70	73.87	1.90	0.32	52.99	61.55	1.98	0.02	44.75	50.40	1.21	0.02
GSH-ST, U/L	14.32	14.80	0.39	0.29	12.02	12.48	0.18	0.28	9.24	9.88	0.42	0.21
MDA, nmol/ml	3.19	2.83	0.20	0.14	4.25	4.43	0.05	0.21	6.23	5.89	0.14	0.07

T-AOC: total antioxidant capacity; SOD: superoxide dismutase; GSH-ST: glutathione s-transferase; MDA: malonaldehyde. CG: control group; SG: shaded group; SEM: standard error; *p*: probabilities.

**Table 6:** Effects of shade on the serum immune parameters in dairy cows.

Immune Parameters	Early-Hot Season				Hot-Season				Late-Hot Season			
	CG	SG	SEM	*p*-Value	CG	SG	SEM	*p*-Value	CG	SG	SEM	*p*-Value
IgA, g/L	0.91	0.85	0.05	0.26	0.60	0.60	0.01	0.91	0.52	0.59	0.04	0.22
IgG, g/L	13.15	12.44	0.33	0.10	6.92	8.17	0.41	0.04	8.66	9.56	0.73	0.33
IgM, g/L	3.70	3.51	0.13	0.20	1.35	1.61	0.09	0.04	0.98	1.06	0.04	0.20
IL-4, g/L	11.75	12.51	0.30	0.07	7.63	8.33	0.20	0.03	5.28	6.38	0.34	0.03
IL-6, g/L	132.06	130.32	1.01	0.16	164.41	165.01	3.02	0.86	181.05	175.79	5.32	0.38

IgA: immunoglobulin A; IgG: immunoglobulin G; IgM: immunoglobulin M; IL-4: Interleukin-4; IL-6: Interleukin-6; CG: control group; SG: shaded group; SEM: standard error; *p*: probabilities.

## Data Availability

The original contributions presented in this study are included in the article. Further inquiries can be directed to the corresponding authors.
